# Differential vulnerability of substantia nigra and corpus striatum to oxidative insult induced by reduced dietary levels of essential fatty acids

**DOI:** 10.3389/fnhum.2012.00249

**Published:** 2012-08-30

**Authors:** Henriqueta D. Cardoso, Priscila P. Passos, Claudia J. Lagranha, Anete C. Ferraz, Eraldo F. Santos Júnior, Rafael S. Oliveira, Pablo E. L. Oliveira, Rita de C. F. Santos, David F. Santana, Juliana M. C. Borba, Ana P. Rocha-de-Melo, Rubem C. A. Guedes, Daniela M. A. F. Navarro, Geanne K. N. Santos, Roseane Borner, Cristovam W. Picanço-Diniz, Eduardo I. Beltrão, Janilson F. Silva, Marcelo C. A. Rodrigues, Belmira L. S. Andrade da Costa

**Affiliations:** ^1^Laboratório de Neurofisiologia, Departamento de Fisiologia e Farmacologia, Centro de Ciências Biológicas, Universidade Federal de PernambucoRecife, Brazil; ^2^Departamento de Bioquímica, Núcleo de Educação Física e Ciências do Esporte, Centro Acadêmico de Vitória da Universidade Federal de PernambucoVitória de Santo Antão, Brazil; ^3^Laboratório de Neurofisiologia, Departamento de Fisiologia, Setor de Ciências Biológicas, Universidade Federal do ParanáParaná, Brazil; ^4^Laboratório de Fisiologia da Nutrição Naíde Teodósio, Departamento de Nutrição, Centro de Ciências da Saúde, Universidade Federal de PernambucoRecife, Brazil; ^5^Laboratório de Ecologia Química, Departamento de Química Fundamental - Centro de Ciências Exatas e da Natureza, Universidade Federal de PernambucoRecife, Brazil; ^6^Laboratório de Investigação em Neurodegeneração e Infecção, Instituto de Ciências Biológicas, Universidade Federal do ParáBelém, Brazil; ^7^Departamento de Bioquímica, Centro de Ciências Biológicas, Universidade Federal de PernambucoRecife, Brazil

**Keywords:** substantia nigra, corpus striatum, oxidative stress, superoxide dismutase, catalase, lipid peroxidation, DHA, neurodegeneration

## Abstract

Oxidative stress (OS) has been implicated in the etiology of certain neurodegenerative disorders. Some of these disorders have been associated with unbalanced levels of essential fatty acids (EFA). The response of certain brain regions to OS, however, is not uniform and a selective vulnerability or resilience can occur. In our previous study on rat brains, we observed that a two-generation EFA dietary restriction reduced the number and size of dopaminergic neurons in the substantia nigra (SN) rostro-dorso-medial. To understand whether OS contributes to this effect, we assessed the status of lipid peroxidation (LP) and anti-oxidant markers in both SN and corpus striatum (CS) of rats submitted to this dietary treatment for one (F1) or two (F2) generations. Wistar rats were raised from conception on control or experimental diets containing adequate or reduced levels of linoleic and α-linolenic fatty acids, respectively. LP was measured using the thiobarbituric acid reaction method (TBARS) and the total superoxide dismutase (t-SOD) and catalase (CAT) enzymatic activities were assessed. The experimental diet significantly reduced the docosahexaenoic acid (DHA) levels of SN phospholipids in the F1 (~28%) and F2 (~50%) groups. In F1 adult animals of the experimental group there was no LP in both SN and CS. Consistently, there was a significant increase in the t-SOD activity (*p* < 0.01) in both regions. In EF2 young animals, degeneration in dopaminergic and non-dopaminergic neurons and a significant increase in LP (*p* < 0.01) and decrease in the CAT activity (*p* < 0.001) were detected in the SN, while no inter-group difference was found for these parameters in the CS. Conversely, a significant increase in t-SOD activity (*p* < 0.05) was detected in the CS of the experimental group compared to the control. The results show that unbalanced EFA dietary levels reduce the redox balance in the SN and reveal mechanisms of resilience in the CS under this stressful condition.

## Introduction

Docosahexaenoic acid (DHA) and arachidonic acid (AA) are long chain polyunsaturated fatty acids (LC-PUFA) which play important roles as critical modulators of brain function under physiological or pathological conditions (Zhang et al., 2011). They are derived from the essential fatty acids (EFA) α-linolenic and linoleic acids, respectively, and can exert opposite effects on brain metabolism (Schmitz and Ecker, 2008). Imbalance in their levels, early in life, and especially DHA deficiency, can decrease anti-inflammatory responses that can induce neurodegeneration (Yavin, 2006; Schmitz and Ecker, 2008). Recent studies using microarray technology have shown that DHA is able to regulate the transcription of many genes related to oxidative stress (OS), cell signaling, and apoptosis (Kitajka et al., 2004; Lapillonne et al., 2004; Yavin, 2006). Consistent with this evidence, it has been demonstrated that DHA protects against peroxidative damage of lipids and proteins in developing and adult brains in experimental models of ischemia-reperfusion (Glozman et al., 1998; Green et al., 2001; Pan et al., 2009; Mayurasakorn et al., 2011) or reduce OS-induced apoptosis of retina photoreceptors (Rotstein et al., 2003). Moreover, the DHA-derived docosanoid, named neuroprotectin D1, protects human retinal pigment epithelial cells from OS (Mukherjee et al., 2004) as well as inhibits brain ischemia-reperfusion-mediated leukocyte infiltration and pro-inflammatory gene expression (Marcheselli et al., 2003).

It is well established that OS is caused by the disequilibrium between the production and detoxification of highly reactive oxygen species (ROS), including singlet oxygen, superoxide anion, and hydrogen peroxide, which can disrupt the redox balance inside cells if not properly neutralized. The superoxide anion is known to induce protein and nucleic acid dysfunction and to initiate lipid peroxidation (LP) (Kohen and Nyska, 2002). Endogenous anti-oxidant mechanisms against superoxides include a series of linked enzyme reactions. The first of these enzymes is superoxide dismutase (SOD; EC1.15.1.1), that converts superoxide anion to hydrogen peroxide (H_2_O_2_), which can be removed by catalase (CAT; EC 1.11.1.6) and/or glutathione peroxidase (GPx; EC 1.11.1.9) (Kohen and Nyska, 2002; Melo et al., 2011).

Neuron response to OS is not uniform in the brain. This differential vulnerability depends on a number of factors including high intrinsic OS, high demand for ROS-based intracellular signaling, low ATP production, mitochondrial dysfunction, and high inflammatory response (Wang and Michaelis, 2010). Strong evidence indicates that OS may be one of the most important mechanisms involved in the etiology and evolution of a number of neurodegenerative diseases (Hashimoto and Hossain, 2011; Thomas and Beal, 2007; Melo et al., 2011). DHA is considered as a potential target for therapeutic intervention in some of these disorders, including Parkinson's Disease (PD), where the dopaminergic neurons of substantia nigra (SN) are especially affected by OS and mitochondrial dysfunction (Jenner et al., 1992; Sayre et al., 2001). In experimental models of PD, for example, it has been shown that the dietary supplementation of DHA may partially restore dopaminergic neurotransmission after 6-hydroxidopamine (6-OHDA)- or 1-methyl-4-phenyl-1,2,3,6-tetrahydropyridine (MPTP)-induced striatal lesions which produce OS (Bousquet et al., 2008; Cansev et al., 2008). Moreover, DHA supplementation is able to increase the SOD activity in the corpus striatum (CS) (Sarsilmaz et al., 2003) as well as significantly decrease cyclooxigenase-2 activity and prostaglandin E2 levels in the SN, decreasing MPTP-induced dopaminergic cell death (Ozsoy et al., 2011). Conversely, combination of successive parity and α-linolenic acid deficient maternal diet reduced the number of dopaminergic neurons in the rat SN pars compacta and ventral tegmental area of adult offspring (Ahmad et al., 2008).

Recent evidence from our laboratory, adopting a two generation model of EFA dietary restriction and stereological assessment, showed a differential vulnerability of two distinct SN dopaminergic cell populations to this type of nutritional insult. In addition to a reduction in the number of dopaminergic neurons in the SN rostro-dorso-medial region, this dietary treatment was able to change body and brain weights, TH protein levels, and the size of these neurons in young animals (Passos et al., 2012). The mechanisms involved in such effects are not yet completely understood. It is well established that under physiological conditions, the SN has unique biochemical features which provide a higher vulnerability to OS (Kidd, 2000) when compared to other brain regions, including the CS (Mythri et al., 2011). The present study was conducted to test the hypothesis that OS can be a potential mechanism involved in the neurodegeneration of SN dopaminergic cells induced by EFA dietary restriction. We tested whether this restriction for one or two generations could induce LP or modify the anti-oxidant activity of SOD or CAT in the SN and CS of rats.

## Materials and methods

All procedures were approved by the Ethics Committee for Animal Research of Federal University of Pernambuco (protocol # 009428/200633), which complies with the “Principles of Laboratory Animal Care” (NIH, Bethesda, USA). Adult female Wistar rats weighing 200–250 g were fed from mating throughout pregnancy and lactation on a control or experimental diets, each containing approximately 400 Kcal 100 g and differing only in the lipid source. The diets were prepared according to Soares et al. (1995) and meet all current nutrient standards for rat pregnancy and growth (Table 1). The control diet contained 50 g/Kg of soybean oil with adequate amounts of saturated, monounsaturated, α-linolenic (6% of total fatty acids) and linoleic (56% of total fatty acids) acids. The experimental diet contained 50 g/Kg of coconut oil (from Babaçu, *Orbignia martiana*) with reduced levels of linoleic and α-linolenic acids and higher levels of saturated (2-fold) and monounsaturated (2.5-fold) fatty acids (Table 2).

**Table 1:** **Diet composition (grams/100g diet)**.

**Ingredients**	**Control diet**	**Experimental diet**
Casein	20.7	20.7
Cellulose	1.8	1.8
Corn starch	46.8	46.8
Sucrose	21.0	21.0
Soyabean oil	5.0	−
Coconut oil	−	5.0
Vitamin mix^*a*^	0.9	0.9
Mineral mix^*b*^	3.7	3.7
D.L-Cystine	0.1	0.1
Butyl hydroxytoluene	0.001	0.001
Kcal/100 g	399.1	400.5

a Vitamin mixture (Rhoster Ind.Com. LTDA. SP. Brazil) containing (m%): folic acid (20); niacin (300); biotin (2); calcium pantothenate 160; pyridoxine (70); riboflavin (60); thiamine chloride (60); vitamin B12 (0.25); vitamin K1 (7.5). Additionally containing (UI%): vitamin A 40.000; vitamin D3 10.000; vitamin E (750).

b Mineral mixture (Rhoster Ind. Com. LTDA. SP. Brazil) containing (m%): CaHP04 (38); K2HP04 (24); CaCO3 (18.1); NaF (0.1); NaCl(7.0); MgO (2.0); MgS04 7H20 (9.0); FeS04 7H20 (0.7); ZnS04 H20 (0.5); MnSO+ H20 (0.5); CuS04 5H20 (0.1); Al2 (S04)3K2S04 24H20 (0.02); Na2SeO3 5H20 (0.001); KCl (0.008).

**Table 2:** **Fatty acid composition of the diets (% of total fatty acids)**.

**Fatty acids**	**Control diet**	**Experimental diet**
8	0.02	3.27
10	0.03	3.95
11	nd	0.07
12	0.20	28.04
13	nd	0.06
14	0.19	19.56
15	0.02	0.02
16	9.27	11.32
17	nd	0.02
18	15.31	0.72
20	0.33	0.16
22	0.51	0.08
23	0.07	0.02
24	0.04	nd
**Total saturated**	**26.01**	**67.29**
16:1	2.72	0.06
18:1n9	9.36	23.51
20:1	0.24	0.16
**Total monounsaturated**	**12.32**	**23.73**
18:2n6	55.36	8.10
18:3n3	6.04	0.49
20:2	0.04	0.06
20:5n3	0.03	nd
22:2n	0.05	0.04
22:6n3	0.13	0.06
**Total polyunsaturated**	**61.65**	**8.75**
18:2n6 /18:3n3	9.17	16.39

nd, not detected. Bold values indicate p < 0.001.

Rat offspring (*n* = 112) were the object of the present study and only males were used for the experimental assays. Litters were culled to six pups on postnatal day 1 and weaned on postnatal day 21. Dams and pups were distributed into two main groups according to the nutritional condition: control (C) and experimental (E) rats. After weaning, pups were separated and fed *ad libitum* the same diet as their respective mothers. First generation (CF1 and EF1) male rats were weighed and evaluated for biochemical parameters related to LP and anti-oxidant markers at 90–110 days. The remaining males and females were allowed to mate to provide the second-generation groups (CF2 and EF2), which were analyzed at 30–42 days. In each group, animals were sampled randomly from different litters, housed three per cage in a room maintained at 22 ± 2°C with 67% relative air humidity and kept on a 12 h light/dark cycle (lights on 6:00 h).

Each experimental day, six animals per group were anesthetized with isofluorane and then decapitated. The regions containing the SN or CS were rapidly dissected in 0.9% (w/v) NaCl solution at 2°C. After weighing, the pooled tissue was homogenized in a 0.9% (w/v) NaCl solution (1:10) at 4°C and centrifuged for 10 min at 1000 g at 4°C for an analysis of LP for the determination of thiobarbituric acid-reactive substances (TBARS) level and for 10 min at 10,000 g at 4°C in order to assess either the total (Cu–Zn and Mn) superoxide dismutase (t-SOD) and catalase enzymatic activities. An aliquot of supernatant was analyzed for total protein content using a bicinchroninic acid protein kit (Sigma-Aldrich, St. Louis, MO).

### Lipid peroxidation

LP was measured by estimating malondialdehyde (MDA) using a thiobarbituric acid (TBA) reaction (TBARS method) according to Ohkawa et al. (1979). In the TBA test reaction, MDA or MDA-like substances and TBA react to produce a pink pigment with maximum absorption at 532 nm. The reaction was developed by the sequential addition of 0.2 mL of 8.1% sodium duodecil sulfate, 1.5 mL of 20% acetic acid (pH 3.5), and 1.5 mL of 0.8% TBA solutions in a boiling water-bath for 30 min to triplicates of supernatants. After tap water cooling, 1.5 mL of n-buthanol / pyridine (15:1 v/v) was added to the sample, centrifuged at 2500 g for 10 min and the organic phase was read at 532 nm using a plate reader. The results were expressed as nmol per mg of protein using a standard curve generated using different concentrations 1,1,3,3-tetramethoxypropane solution. The control SN and CS samples were incubated in a 30 μM sodium nitroprusside (SNP) solution for 45 min before the assay and used as positive controls for LP.

### Superoxide dismutase (SOD) assay

Assessment of total SOD (t-SOD) enzymatic activity was performed according to Misra and Fridovich, (1972) at 25°C. Triplicates of SN or CS supernatants (100 μL) were previously incubated in a water bath at 37°C and then added to 880 μL of 0.05% sodium carbonate solution pH 10.2 in 0.1 mM EDTA. The reaction was developed by adding 20 μL of 30 mM epinephrine (in 0.05% acetic acid). The absorbance was measured at 480 nm for 4 min. One unit of t-SOD was defined as the enzyme amount causing 50% inhibition of epinephrine oxidation. Tissue t-SOD enzymatic activity was also expressed as units per milligram of protein (U/mg protein). Positive controls were obtained incubating control homogenate samples of SN and CS in a 30 μM SNP solution for 45 min before the enzymatic assay.

### Catalase (CAT) assay

CAT activity was measured according to Aebi (1984). The rate constant k of H_2_O_2_ decomposition under our experimental conditions of temperature (~20°C) and pH (7.0) was determined to be 4.6 × 10^7^ by measuring the absorbance changes per minute, for 4 min. The enzymatic activity was expressed as the H_2_O_2_ consumed in nM/min/mg protein. Positive controls for catalase activity were obtained by incubation of SN and CS homogenates of the control group in increasing concentrations of H_2_O_2_ (3.156 to 100 μM) for 30 min at 37°C before the enzymatic assay.

### Statistical analysis of oxidative stress parameters and body weight

All biochemical experiments were carried out in triplicate and repeated at least twice. Six animals from three litters per group were used each time. A total of 38 and 58 animals were used in the F1 and F2 generations, respectively. Biochemical data of TBARS levels, t-SOD, and catalase enzymatic activity were plotted using GraphPad Prism 5.0 software and the statistical analysis was performed using ANOVA followed by Tukey as the *post-hoc* test or Student's *t*-test in some cases. The analysis of body weight was carried out using unpaired Student's *t*-test. Differences were considered significant when *p* < 0.05.

### Fluoro jade C (FJC) assay

Considering our recent evidence that a loss of SN dopaminergic cells is induced by EFA dietary restriction for two generations (Passos et al., 2012), FJC, a polyanionic fluorescein derivative, was applied to examine signals of neurodegeneration. It has been shown that this protocol specifically labels damaged neurons and not glial cells in the SN and CS (Bian et al., 2007; Ehara and Ueda, 2009) when these regions are submitted to certain types of insult, especially under conditions that induce OS (Ehara and Ueda, 2009; Li et al., 2009; Yang et al., 2011).

Animals from the F1 and F2 groups (*n* = 6/group) were anesthetized with a sodium pentobarbital solution (100 mg/kg, i.p. Sigma-Aldrich, St. Louis, MO), perfused with a 0.9% NaCl solution, followed by 4% paraformaldehyde in a phosphate buffered saline (PBS), pH 7.4. The brains were post-fixed in the same fixative for two hours, rinsed in a phosphate buffer (PB) and subsequently cryoprotected in solutions of 10, 20, and 30% sucrose in PB. Brain blocks were serially cut on a freezing microtome (Leitz Wetzlar) into 50 μm-thick sections in the parasagittal plane. All sections were collected serially in PB and arranged in six series. The Atlas of Paxinos and Watson (1986) was used to delimit cytoarchitectonic regions of interest. Sections of one series per animal were mounted on gelatin-coated slides, air-dried, and subjected to FJC staining according to Ehara and Ueda (2009). Slides were immersed in a 1% NaOH solution (in 80% ethanol) for 5 min, rinsed for 2 min in 70% ethanol, and for 2 min in distilled water, and then incubated in 0.06% potassium permanganate solution for 5 min. After water washing (2 min), the slides were immersed in a FJC solution (0.0001%) in 0.1% acetic acid for 10 min followed by washing in distilled water. The slides were air-dried on a slide warmer at 50°C for 30 min, cleared in xylene, cover slipped with Entellan (Merck). As a positive control for FJC labeling we used brain sections of rats previously treated with the mitochondrial toxin 3-Nitropropionic Acid (3-NP) which induces striatal neurodegeneration. The animals treated with 3-NP were from another study not related to the present work. As a better positive control for FJC labeling in the SN, we used also brain sections of animals which previously received intracerebral injections of pilocarpine in order to induce epilepticus status. The number of FJC-positive neurons was analyzed in the CS and SN in six animals of C and EF2 groups at the stereotaxic coordinate identified as corresponding approximately to lateral 1.9 mm (plate 81) according to Paxinos and Watson (1986).

Double fluorescence staining against FJC and tyrosine hydroxylase in brain sections of two EF2 animals was achieved by the method described by Ehara and Ueda (2009). Tissue sections were incubated first with blocking solution containing 1% BSA, 0.3% triton X-100 for 60 min and then with rabbit polyclonal anti-TH antibody (1:500; Millipore) for 24 h at 4°C. The sections were washed three times in phosphate buffer (PB) 0.1 M, pH 7.4, and incubated for 4 h with Rhodamine-conjugated 546-labeled anti-rabbit IgG (1:600; Jackson). After washing twice in PB, they were mounted onto gelatin coated slides and dried at 50°C for 30 min. The samples were rehydrated for 1 min, incubated in 0.06% potassium permanganate solution for 5 min, and then rinsed for 1 min in distilled water followed by FJC (0.0001% dissolved in 0.1% acetic acid) for 30 min. After rinsed in distilled water, the sections were dried at 50°C for 20 min, clearead in xylene for 1 min and coverslipped with Entellan. Fluoro-Jade C and TH in the CS and SN were analyzed using an epifluorescence microscope (Leica, DM LB).

### Fatty acid determination in the corpus striatum and midbrain

The fatty acid profiles of CS and midbrain phospholipids were assessed in F1 groups at 95 days and F2 groups at 35 days of age. The pups (*n* = 6/group) were decapitated and the regions containing the CS or midbrain were rapidly dissected in an ice bath. The tissues were homogenized in a 50 mM Tris-HCl buffer (pH = 7.4) with EGTA and centrifuged for 30 min at 28,000 g at 4°C. The pellets were immediately re-suspended in 50 mM Tris-HCl buffer (pH = 7.4). The total lipids of CS or midbrain homogenates were extracted according to Folch et al. (1957). The phospholipids were then separated by means of a Sep-Pak procedure (Juaneda and Rocquelin, 1985) and transmethylated (Berry et al., 1965). These samples were analyzed using a Shimatzu GC apparatus equipped with a flame ionization detector and HP-inowax 20 M) capillary column (30 m × 0.32 mm × 0.3 μm). The column temperature was initially 40°C for 1 min, then increased to 150°C by 55°C/min, and finally increased to 220°C by 1.7°C/min. The injector and detector temperatures were 200 and 220°C, respectively. Hydrogen was used as the carrier gas at a flow rate of 1.0 mL/min; injection was in split-less mode and the injection volume was 1.0 μL of the sample isoctane extract. A standard fatty acid methyl ester mixture (Supelco™, 37 Component FAME mix, USA) was used to identify the fatty acid methyl esters by their retention time. Fatty acid data were expressed as percentage of total peak area. Data are expressed as the mean ± standard deviation (SD). Differences between the groups were analyzed by Student's *t*-test and considered significant at *P* < 0.05.

## Results

Data on body weights of F1 and F2 groups are presented in Table 3. Adult animals of the EF1 group and young animals of the EF2 group showed significantly lower body weights when compared to the control (*p* < 0.05).

**Table 3:** **Body weights of F1 and F2 animals**.

**Groups**	**Body Weight (g)**
CF1	402.54 ± 40.04 (n = 38)
EF1	376.97 ± 36.92^**^ (n = 43)
CF2	79.65 ± 14.87 (n = 33)
EF2	71.91 ± 10.09^*^ (n = 43)

Values are expressed as Mean ± SD.

* P < 0.05

** P < 0.01 Unpaired Student's t-test.

### Corpus striatum and midbrain fatty acid profile

Table 4 shows the midbrain fatty acid profile of F1 generation adult animals and Table 5 combines data of midbrain and CS fatty acids of the F2 generation young animals raised under either control or experimental diets. As can be observed, the midbrain phospholipids from the EF1 and EF2 groups exhibit, respectively, 28 and 50% lower DHA levels (22:6n–3) as compared to their control groups. DHA levels were also lower in the EF2-CS phospholipids (~50%) when compared to control. The reduced levels of DHA in both EF1 and EF2 groups was accompanied by a significant increase in the docosapentanoic fatty acid (DPA; 22:5n6) contents (2-tail *t*-test, *P* < 0.001). On the other hand, the values for AA (20:4n–6) did not differ between both groups of F1 or F2 generations. With respect to saturated and monounsaturated fatty acids, the presence of coconut oil in the maternal diet significantly increased the levels of palmitic (16:0), stearic (18:0), palmitoleic (16:1), and oleic (18:1n9) acids (2-tail *t*-test, *P* < 0.01) in the EF2 midbrain phospholipids.

**Table 4:** **Fatty acid composition (% of total) in midbrain phospholipids of F1 generation groups raised on Control or Experimental diets**.

**Fatty acid**	**Midbrain**	
	**Control diet**	**Experimental diet**
C16	16.41 ± 1.9	15.85 ± 0.81
C16:1	0.96 ± 0.34	1.10 ± 0.28
C18	22.47 ± 1.63	23.99 ± 1.87
C18:1n9	24.55 ± 0.96	24.64 ± 2.00
C20	0.74 ± 0.13	0.91 ± 0.10
C20:1	2.04 ± 0.21	2.83 ± 0.12
C20:4n6	8.76 ± 0.32	8.73 ± 0.30
C20:3n6	0.45 ± 0.30	0.35 ± 0.24
C22	0.97 ± 0.53	0.80 ± 0.11
C23	3.11 ± 0.51	3.10 ± 0.52
C22:5n6	1.03 ± 0.74	3.16 ± 0.75^***^
C22:6n3	14.41 ± 1.81	11.25 ± 0.69^**^
C24:1n	2.24 ± 0.52	1.07 ± 0.18

Values are expressed as means ± SD.

** p < 0.01

*** p < 0.001.

**Table 5:** **Fatty acid composition (% of total) in Corpus Striatum and Midbrain membrane phospholipids of F2 generation groups raised on Control or Experimental diets**.

**Fatty acid**	**Corpus Striatum**		**Midbrain**	
	**Control diet**	**Experimental diet**	**Control diet**	**Experimental diet**
C16	17.99 ± 1.04	21.74 ± 0.74	16.51 ± 1.90	24.09 ± 0.27^*^
C16:1	0.73 ± 0.10	0.73 ± 0.12	0.64 ± 0.04	0.85 ± 0.03
C17	Nd	nd	0.16 ± 0.00	0.20 ± 0.02
C18	25.74 ± 0.25	25.20 ± 0.51	24.88 ± 0.67	29.09 ± 0.37^*^
C18:1n9	17.07 ± 0.34	15.18 ± 0.69	16.38 ± 0.25	19.21 ± 0.21^*^
C18:2n6t	nd	nd	0.74 ± 0.17	0.65 ± 0.22
C20:1	nd	nd	0.62 ± 0.10	0.60 ± 0.05
C20:4n6 (AA)	14.08 ± 0.27	13.12 ± 0.34	13.74 ± 0.83	14.93 ± 0.48
C23	3.88 ± 0.13	3.46 ± 0.18	3.98 ± 0.20	3.52 ± 0.19
C22:5n6	1.54 ± 0.06	9.60 ± 0.26^**^	1.49 ± 0.11	9.39 ± 0.31^**^
C22:6n3 (DHA)	19.23 ± 0.88	9.48 ± 0.84^**^	19.70 ± 0.69	8.70 ± 0.73^**^

Values are expressed as means ± SD.

* p < 0.01

** p < 0.001

nd, not detected.

### Lipid peroxidation and T-SOD enzyme activity in adult animals of F1 generation

**Figure 1:**
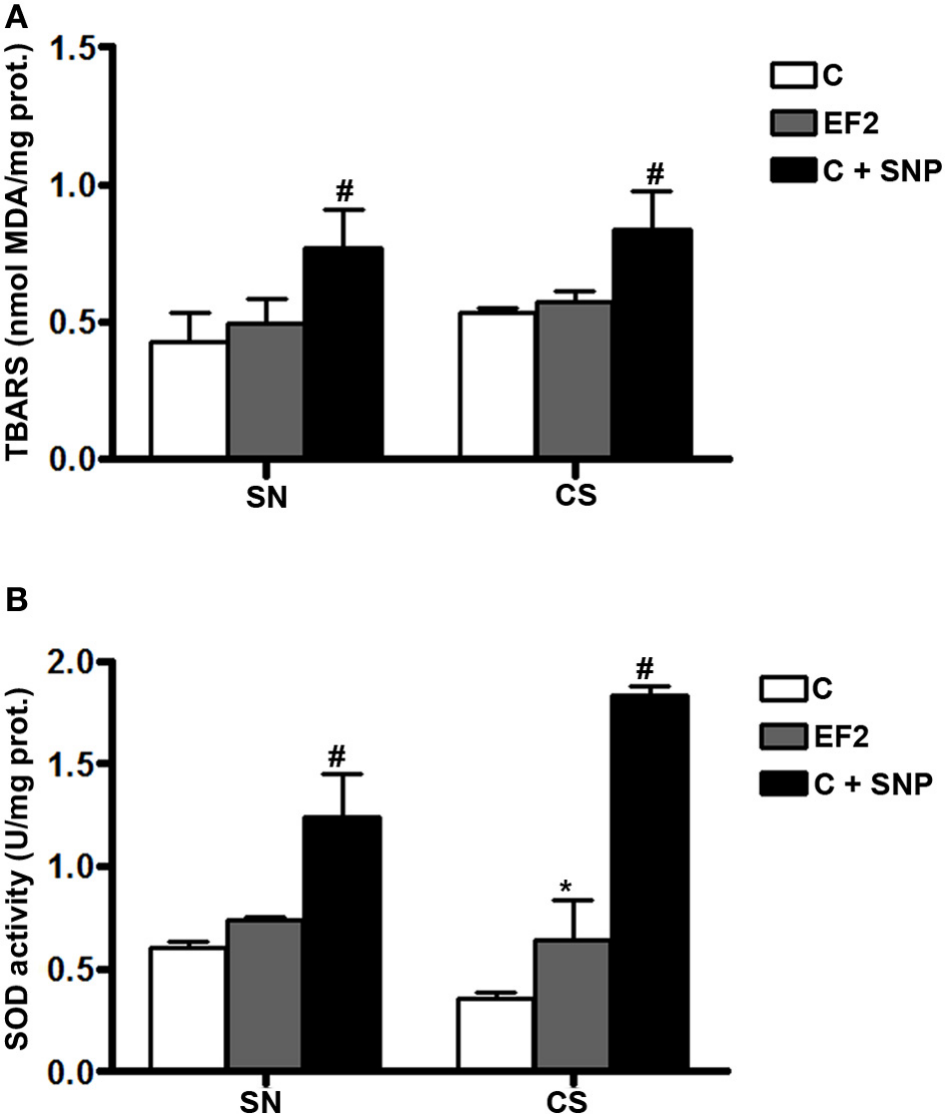
**Thiobarbituric acid-reactant substances (TBARS) levels (A) and total superoxide dismutase (t-SOD) activities (B) in the pool of Substantia Nigra and Corpus Striatum from first generation adult rats fed essential fatty acid restricted diet and controls (*n* = 12 per group)**. ^*^P < 0.05 compared to control group. Treatment of control homogenates with sodium nitroprusside (SNP) was used as positive control in all the experiments. #*P* < 0.001 compared to control or EF1 groups.

Biochemical results of the F1 groups are summarized in Figure 1. As expected, LP (measured as TBARS levels) was found to be significantly increased in SN (0.770 ± 0.136 nmol MDA/mg protein) and CS (0.834 ± 0.140 nmol MDA/mg protein) homogenates of CF1 group previously treated with 30 μM SNP, compared to the control condition (0.425 ± 0.105 and 0.532 ± 0.015 nmol MDA/mg protein for SN and CS, respectively; *P* < 0.001). However, TBARS levels in both regions were not modified in rats fed on the experimental diet (0.494 ± 0.089 and 0.570 ± 0.038 nmol MDA/mg protein for SN and CS, respectively) when compared to the control animals (Figure 1A). Consistent with these results, a significant increase in the t-SOD enzyme activity was observed in the EF1 group (*P* < 0.01) either in the SN (0.735 ± 0.020 U/mg protein) or CS (0.640 ± 0.192 U/mg protein) compared to the control condition not submitted to pre-treatment with SNP (0.606 ± 0.028 and 0.355 ± 0.034 U/mg protein for SN and CS, respectively). As can be observed, the SNP treatment used as a positive control, significantly increased SOD activity in the SN (1.241 ± 0.206 U/mg protein) and CS (1.832 ± 0.046 U/mg protein).

### Lipid peroxidation, T-SOD, and CAT enzyme activities in young animals of F2 generation

**Figure 2:**
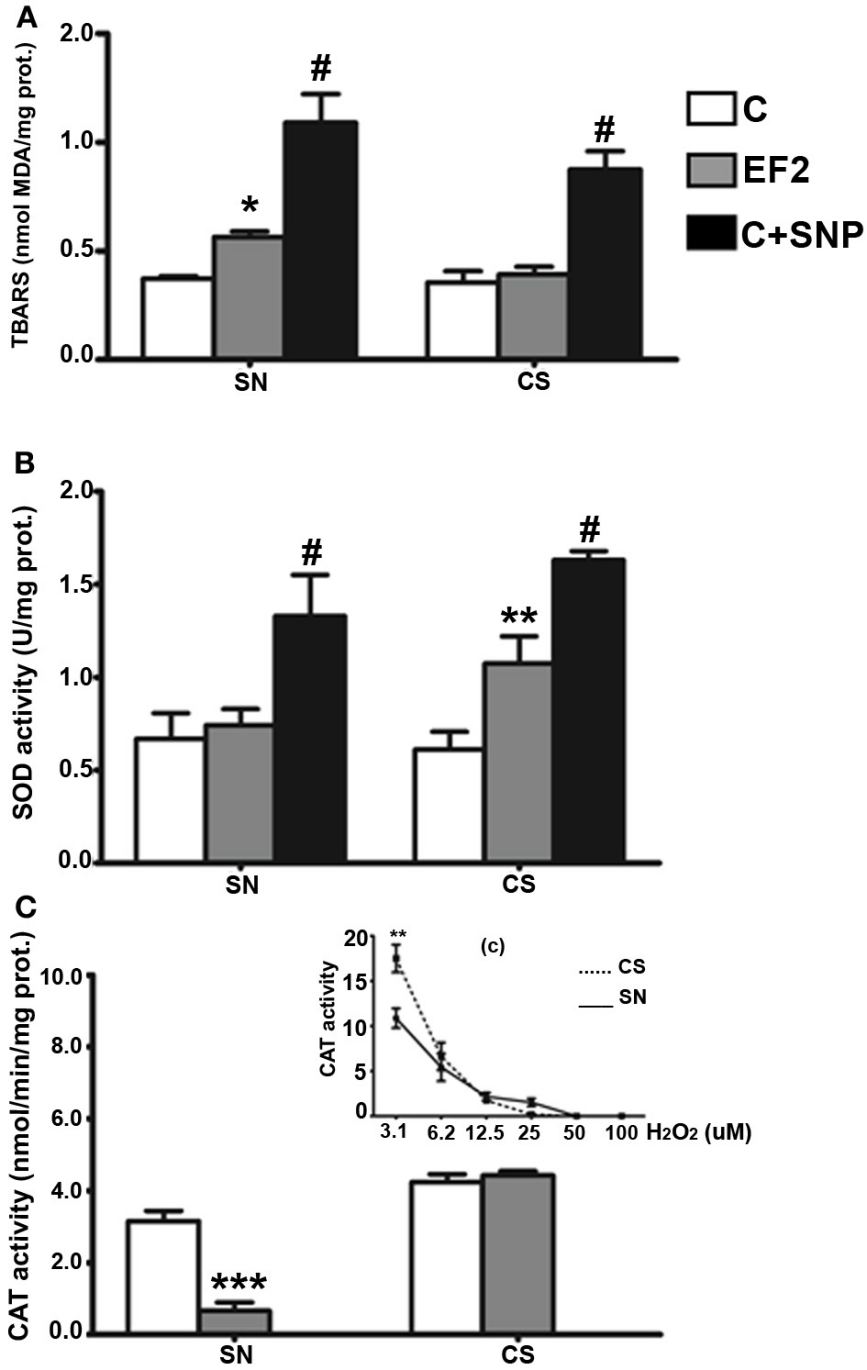
**Thiobarbituric acid-reactant substances (TBARS) levels (A), total superoxide dismutase (t-SOD) activities (B), and catalase (CAT) activities (C) in the pool of Substantia Nigra and Corpus Striatum from young rats fed an essential fatty acid restricted diet over two generations and respective controls (*n* = 12 per group)**. ^*^P < 0.05; ^**^P < 0.001 compared to control group. Treatment of control homogenates with sodium nitroprusside (SNP) was used as positive control for TBARS and t-SOD in all the experiments. The insert (c) in the panel **(C)** shows the H_2_O_2_ concentration-dependent manner of the CAT activity in the SN and CS control homogenates obtained as positive controls. #*P* < 0.0001 compared to control or EF2 groups.

In young animals of the F2 generation, distinct effects were induced by the experimental diet in the two regions analyzed. Evidence of LP, assessed by a significant increase in TBARS levels, was detected in the SN of EF2 group (0.564 ± 0.02 nmol MDA/mg protein) in comparison with the control group (0.372 ± 0.01 nmol MDA/mg protein, *P* < 0.05). The magnitude of LP induced by the experimental condition in the SN is about 50% less than that obtained by using 30 μM SNP (1.330 ± 0.220 nmol MDA/mg protein). No difference between the EF2 (0.354 ± 0.005 nmol MDA/mg protein) and the C (0.391 ± 0.083 nmol MDA/mg protein) groups was found in the CS (Figure 2A). A significant increase in t-SOD enzyme activity was found in the CS of the EF2 group (1.074 ± 0.145 U/mg protein) compared to the control group in the absence of pre-treatment with SNP (0.610 ± 0.096 U/mg protein, *P* < 0.01). Nevertheless, the increase in SOD activity in the EF2 group CS was smaller than that induced by 30 μM SNP in the C group (1.633 ± 0.046 U/mg protein). No difference between the groups was detected for t-SOD activity in the SN (0.741 ± 0.087 and 0.667 ± 0.138 U/mg protein for the EF2 and C groups, respectively) as shown in the Figure 2B. On the other hand, the CAT activity was significantly reduced in the SN of the EF2 group (0.652 ± 0.238 nmol/min/mg protein) compared to the control group (3.159 ± 0.279 nmol/min/mg protein in the control; *P* < 0.001). No difference between the groups was detected in CAT activity in the CS (4.339 ± 0.217 nmol/min/mg protein and 4.420 ± 0.125 nmol/min/mg protein for the EF2 and C groups, respectively) as shown in Figure 2C. The insert in the Figure 2C shows the H_2_O_2_ concentration-dependent manner of the CAT activity in the SN and CS control homogenates obtained as positive controls. As can be observed, at lower concentrations of H_2_O_2_, the CAT activity is significantly greater in the CS as compared to SN (*p* < 0.05) but this difference disappears at higher concentrations.

### Fluoro jade C and tyrosine hydroxilase labeling

**Figure 3:**
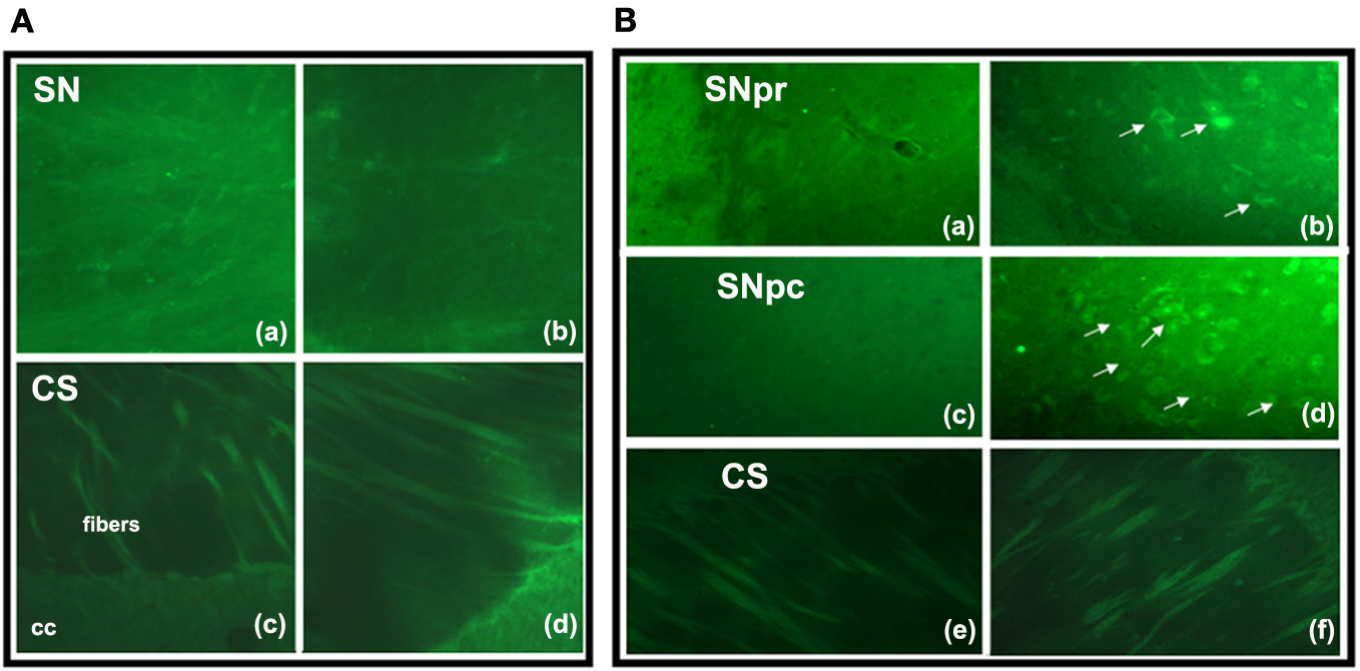
**Fluoro-Jade C staining in brain parasagittal sections of F1- (panel A) or F2- (panel B) generation groups at the level of Substantia nigra (SN) or Corpus Striatum (CS)**. No labeling was detected in cell bodies or processes of SN **(Aa, Ab)** and CS **(Ac, Ad)** in adult animals of F1 generation. However, FJC positive cell bodies and processes were detected in the SN pars reticulata **(Bb)** and pars compacta **(Bd)** of EF2 group while no labeling in these regions was seen in the controls **(Ba, Bc)**. No FJC labeling was detected in cell bodies of the CS in the EF2 **(Bf)** or control **(Be)** groups. A slight and non-specific labeling was seen in regions rich in myelin such as cerebral peduncle (cp), corpus callosum (cc), or myelinated fibers crossing the CS.

Fluoro-Jade C-positive cell bodies were not detected in the SN or CS in the groups (6 animals/group) of F1 generation (Figure 3A). In the SN of the EF2 group, several FJC-positive cells were seen either in the pars compacta or in the pars reticulata while no labeling was detected in cell bodies of the CS in all animals (*n* = 6) analyzed (Figure 3B). In the EF2 group (*n* = 6), the number of FJC-positive cells distributed in the *pars compacta* and *pars reticulata* at the middle level of SN changed from 59 to 70 cells and the average number was estimated as 63.8 ± 6.4 cells.

**Figure 4:**
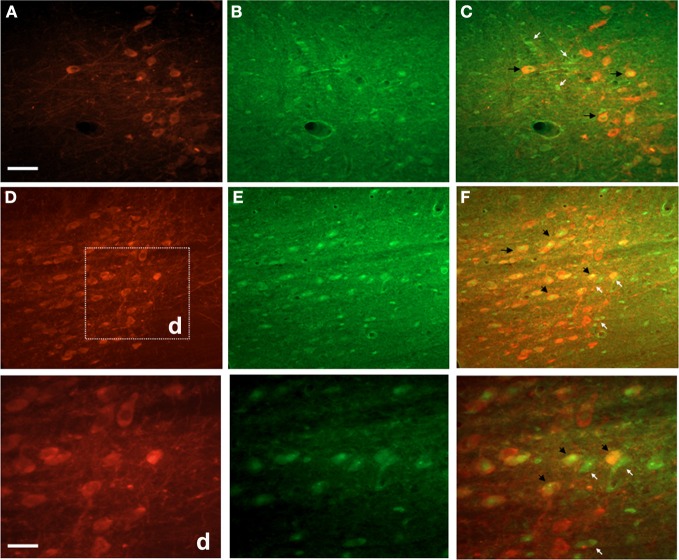
**Photographs of epifluorescence microscopy showing SN sections from a representative EF2 animal subjected to TH immunostaining followed by Fluoro-Jade C staining**. Examples of single (FJC; yellow arrows) or double (TH + FJC; black arrows) labeled cells can be seen either in the SN *pars reticulata* (**A,B**, and **C**) or in the *pars compacta* (**D,E**, and **F**). High magnification of the region (d) is shown in the bottom panel. Scale bar of **A** = **B,C,D,E**, and **F** represents 60 μm while the scale bar of bottom panel represents 20 μm.

**Figure 5:**
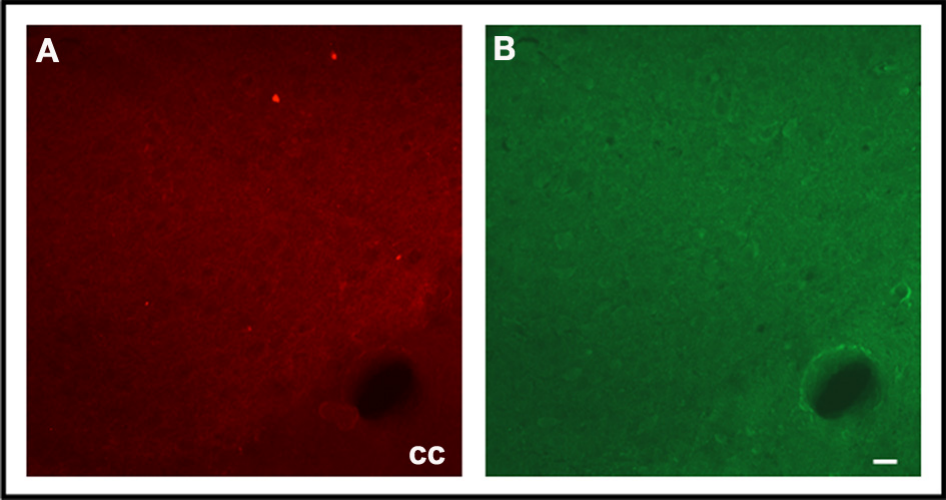
**Photographs of epifluorescence microscopy showing CS sections from a representative EF2 animal subjected to TH immunostaining (A) followed by Fluoro-Jade C staining (B)**. Note the absence of FJC-positive cells surrounded by TH-positive neuronal terminals. cc = corpus calosum, Scale bar = 40 μm.

Double fluorescence staining for FJC and TH of a representative EF2 animal is shown in the Figure 4. As can be seen, signals of degeneration were detected in SN dopaminergic and non-dopaminergic neurons either in the *pars reticulata* or in the *pars compacta*. Nevertheless, no staining for FJC was found in cell bodies surrounded by TH-positive neuronal terminals in the CS, confirming data obtained using single labeling for FJC (Figure 5).

## Discussion

The current study investigated whether a dietary restriction of both linoleic and α-linolenic fatty acids for one or two generations could affect the redox balance in the SN and CS. We hypothesized that OS could be a potential mechanism involved in the loss of dopaminergic cells previously demonstrated (Passos et al., 2012). Our data showed signals of degeneration in SN dopaminergic and non-dopaminergic neurons and indicated a differential vulnerability of SN and CS to oxidative insult induced by two generations of EFA dietary restriction.

### Repercussion of dietary treatment on body weight

The significant lower body weight gain of adult EF1 and young EF2 animals is in agreement with previous studies using coconut oil as the only source of dietary lipids (Deuel et al., 1954; Soares et al., 1995; Borba et al., 2010). Regarding this effect, this type of dietary treatment has been associated with dysfunction of growth hormone regulation (Soares et al., 1995). Moreover, it has been reported that coconut oil can reduce body weight due to high saturated medium chain fatty acids (8:0–14:0) turnover rates, which are predominant in its lipid profile. Such effect seems to be independent of essential fatty acid deficiency (Hargrave et al., 2005).

### Midbrain and corpus striatum fatty acid profile

It has been demonstrated that a diet containing coconut oil as the only source of lipids depletes DHA in the brain more than a fat free diet, even for a short-term treatment, especially due to the diet's high content of saturated fatty acids (Ling et al., 2010). In the present study, the experimental diet based on coconut oil significantly reduced DHA levels about 28 and 50% in the midbrain phospholipids of the EF1 and EF2 groups, respectively, as compared to their controls. The DHA depletion was accompanied by a significant increase in DPA levels, which reinforces the condition of DHA deficiency. On the other hand, despite containing 8% linoleic acid (about 30% of recommended minimal dietary requirement for rodents (Bourre et al., 1990), the experimental diet did not modify the AA levels in either region of EF2 group. These results agree with other studies, indicating that AA is more tightly controlled than DHA in the central nervous system and that its brain concentrations are less vulnerable to limitations in the supply of precursor than other organs (Bourre et al., 1990; Brenna and Diau, 2007; Igarashi et al., 2009; Ling et al., 2010). In fact, recent evidence has indicated that even when using a diet containing 2.3% linoleic acid for 15 weeks, starting at weaning, the brain AA concentration is reduced by only 28%, while a 74% reduction has been observed in the liver of the same rats (Igarashi et al., 2009). Thus, in addition to DHA deficiency, our dietary treatment was able to increase AA/DHA ratio in the fatty acid profile of SN and CS phospholipids.

### Repercussion of dietary treatment on lipid peroxidation and enzymatic anti-oxidant activity

It has been established that an imbalance in the AA/DHA ratio and especially DHA deficiency can decrease anti-inflammatory and anti-oxidant responses and induce cellular damage in different classes of neurons (Yavin, 2006; Schmitz and Ecker, 2008). An inverse relation between the number of some brain neurons and increasing ratios of n–6/n–3 EFAs in the maternal diet has been also recently reported (Tian et al., 2011). In the present study, an increase in the t-SOD activity observed in the SN and CS of the EF1 group was able to protect these regions from membrane LP measured as TBARS levels. The absence of FJC labeling in neuronal cell bodies of both brain regions reinforces these results, considering the efficacy of this reagent in detecting signals of neurodegeneration induced by conditions of OS, such as ischemia (Yang et al., 2011), glutamate excitotoxicity (Ehara and Ueda, 2009) or dopaminergic lesions induced by 6-OHDA (Ehara and Ueda, 2009) or MPTP (Bian et al., 2007; Li et al., 2009).

EFA dietary restriction over two generations, which induced a more expressive DHA deficiency in midbrain phospholipids (~50%) and AA/DHA ratio (~2), was able to provoke LP and impaired the anti-oxidant responses at least in SOD and CAT enzymes in the SN of the EF2-group as compared to the control. Such results are consistent with recent evidence of the protective action of DHA dietary supplementation on SN cell populations under experimental conditions that induce OS, such as MPTP (Ozsoy et al., 2011). The lack of efficient t-SOD reactivity and the expressive reduction in the CAT activity observed in the EF2 group shows the vulnerability of SN to conditions that reduce DHA availability during the critical period of brain development. Studies on rats or human SN have indicated a progressive decrease in the activity of several anti-oxidant enzymes including SOD and CAT during physiological brain aging (Kolosova et al., 2003; Venkateshappa et al., 2012). The present findings in the EF2 young animals corroborate our initial hypothesis indicating that a decreased anti-oxidant function can be a potential mechanism by which long-term EFA dietary restriction induces loss of SN dopaminergic neurons (Passos et al., 2012). Thus, increased levels of OS in the young brain might act synergistically with other deleterious effects induced by DHA deficiency, accelerating the degenerative profile of SN. The FJC staining in the SN of EF2 animals reinforces these data, demonstrating the presence of neuronal damage in several dopaminergic neurons either in the *pars compacta* or in the *pars reticulata*. Moreover, we also detected signals of degeneration in non-dopaminergic cells at the same regions of SN, suggesting that the oxidative insult induced by EFA dietary restriction affects neuronal populations with distinct neurochemical profile.

In contrast to the effects detected in the SN and despite a similar DHA deficiency, we did not observe LP or anti-oxidant dysfunction in the CS of the EF2 young rat brains, when compared to their respective controls. In support of this biochemical data, we did not find FJC-positive cell bodies in parasagittal or transversal sections of this nucleus. These findings reinforce some early and recent evidence in human and experimental animals that this region is more resistant than SN under physiological (Kolosova et al., 2003; Venkateshappa et al., 2012) or pathological conditions where SN dopaminergic neurons are affected (Floor and Wetzel, 1998; Mythri et al., 2011). The significant increase in the t-SOD activity in the CS of the EF2 animals indicates that this region has differential compensatory means which can be triggered from the insult induced by DHA deficiency. It is noteworthy that under normal conditions, dietary DHA supplementation, even for a short period (30 days), is able to increase the t-SOD activity in the CS of adult rats, which has been suggested as a potential regulatory action of this LC-PUFA on this enzyme (Sarsilmaz et al., 2003). If this is the case, our findings suggest that such action could be activated even under conditions of 50% DHA depletion in the CS phospholipids. A differential reactivity of CS under OS conditions was also recently reported: in animals injured with 6-OHDA, the dopamine turnover is significantly increased in this nucleus by fish oil supplementation (Delattre et al., 2010).

The increased t-SOD activity in the CS was not accompanied by a similar CAT reaction, which did not change its activity as compared to the control condition. These enzymes play complementary activities in the anti-oxidative defense system, considering that the H_2_O_2_ generated by SOD activity is the substrate for CAT. Thus, the absence of LP in the CS suggests that other anti-oxidant mechanisms involved in the degradation of H_2_O_2_ could be implicated in the relative resistance of this nucleus. An expressive increase in the total glutathione levels and in the glutathione peroxidase activity associated with glial cell proliferation has been found in the CS and frontal cortex of human postmortem PD brains (Mythri et al., 2011). Although future studies need to be carried out in order to address this issue in our experimental model, preliminary results of our group indicate that the glial cell reactivity might be also implicated in the lower vulnerability of CS to oxidative insult described herein.

Despite the resistance of CS to OS under the present experimental conditions, we cannot discard potential effects of EFA dietary restriction on other parameters involving neuroprotection in the nigrostriatal system. The anti-oxidant parameters here investigated under conditions of DHA deficiency probably are not working alone. A recent study demonstrated that a relatively short-term feeding of an α-linolenic acid-restricted diet was able to lower the DHA content and the brain derived neurotrophic factor (BDNF) levels in the mouse striatum, while two other brain regions were not affected (Miyazawa et al., 2010). Conversely, when DHA is supplemented in the diet, CS strongly reacts to OS induced by MPTP, increasing the synthesis of BDNF more than in control conditions (Bousquet et al., 2009). The disrupted relation between OS and neurotrophin availability could be involved in behavioral or neurochemical effects observed in animals or human beings submitted to EFA dietary restriction (Fedorova and Salem, 2006; Kuperstein et al., 2008).

## Conclusion

The present data shows the importance of adequate dietary levels of EFA to maintain an effective redox balance in the SN. Our results demonstrate that LP associated with an impaired anti-oxidant response increases the vulnerability of SN dopaminergic and non-dopaminergic neurons to degeneration induced by long-term EFA dietary restriction. These results reinforce the hypothesis that this dietary treatment increases the risk of certain neurological disorders. The data also demonstrate that biological mechanisms of resilience can be activated in the CS even under a similar condition of DHA deficiency.

### Conflict of interest statement

The authors declare that the research was conducted in the absence of any commercial or financial relationships that could be construed as a potential conflict of interest.
